# Modeling and Optimization of Multiple Unmanned Aerial Vehicles System Architecture Alternatives

**DOI:** 10.1155/2014/189679

**Published:** 2014-07-20

**Authors:** Dongliang Qin, Zhifei Li, Feng Yang, Weiping Wang, Lei He

**Affiliations:** College of Information System and Management, National University of Defense Technology, Changsha, Hunan 410073, China

## Abstract

Unmanned aerial vehicle (UAV) systems have already been used in civilian activities, although very limitedly. Confronted different types of tasks, multi UAVs usually need to be coordinated. This can be extracted as a multi UAVs system architecture problem. Based on the general system architecture problem, a specific description of the multi UAVs system architecture problem is presented. Then the corresponding optimization problem and an efficient genetic algorithm with a refined crossover operator (GA-RX) is proposed to accomplish the architecting process iteratively in the rest of this paper. The availability and effectiveness of overall method is validated using 2 simulations based on 2 different scenarios.

## 1. Introduction

Unmanned aerial vehicle (UAV) systems, which were developed for military purpose originally, are currently in limited use for public service missions worldwide. UAV systems come in all sizes, and although most are for military applications, they have already been used in civilian activities. They can operate in areas where it would be irresponsible to expect pilots to fly, for example, low level, night flights over the Arctic Ocean, flights over regions where there is low level strife. Here, the disaster management supported by UAVs is taken for demonstrating. UAV systems have potential to augment the effectiveness of disaster response dramatically based on their excellent performance in dull, dirty, or dangerous environment. Disaster relief and emergency response missions could be a fifth category in the civil uses of UAVs systems [1]; they are expected to be “force multipliers” in disaster response just as in achieving military missions. Disaster response consists of various types of tasks, that is, warning/evacuation, search and rescue, providing immediate assistance, assessing damage, continuing assistance, and the immediate restoration of infrastructure. Huge difference exists between different tasks in the degree of urgency and difficulty. The effectiveness of disaster response depends on the coordination of these tasks.

Studies on UAVs for disaster monitoring have been conducted by several researchers [2, 3]. Also, some problems related to multiplatforms (UAVs included) collaborative disasters monitoring have conducted by BAI GuoQing [4, 5].

In this paper, we focus on the modeling and optimization of multiple UAVs system architecture to maximize mission performance. Driven by the tasks to be conducted in given mission, the UAVs system architecture model can be treated as a solution of corresponding task assignment problem.

## 2. Architecture and Architecting

**Figure 1:**
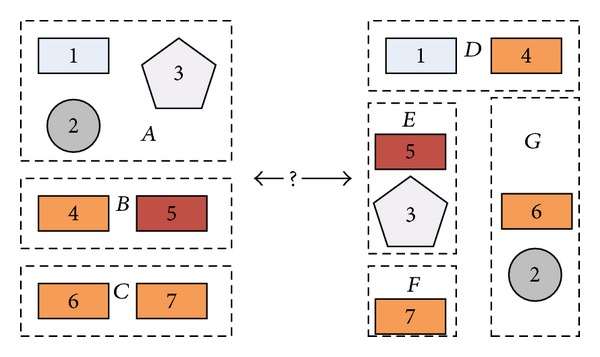
System architecture process.

The first researcher to formalize the concept of “systems architecting” is Rechtin, and his book with Maier is the best introduction to this field [6]. More formally, Crawley defines architecture as “an abstract description of the entities of a system and the relationships between those entities.” However, a more precise definition is given by Crawley in MIT Engineering Systems Division as follows: “the embodiment of a concept: the allocation of physical/informational function to elements of form, and the definition of interfaces among the elements and with the surrounding context [7].” This precise definition can be expressed briefly as function-to-form mapping where the function executed by the different subsystems and the form is the relationship among them. The allocation of physical or informational elements of function into elements of form is one of the earliest and most important architectural decisions that have to be made. The concept of system architecture can be illustrated as in Figure 1. There are 7 different subsystems for architecting, and every subsystem can execute certain function. It is supposed that there are two alternatives to be selected. The function-to-form mapping of left alternative is {*A*: {1, 2, and 3},* B*: {4, 5},* C*: {6, 7}}. Function 1, function 2, and function 3 are mapping to form* A*; function 4 and function 5 are mapping to form* B*; function 6 and function 7 are mapping to form* C*. The mission can be accomplished by the combination of form* A*, form* B*, and form* C*. The right alternative is another function-to-form mapping which can also accomplish the mission. Then, the problem is which alternative to be selected as the ultimate architecture. The selecting process is usually based on the mission performance. This is the essence of the system architecting.

The system architecture can be comprehended in two aspects. Firstly, it should tell us “what the system is” or the set of tangible elements that the system is composed of. Secondly, it should tell us “what the system does” in order to provide value to the stakeholders. System architecting is the process of creating system architecture.

The system architecting process can essentially be seen as a decision making process where the decisions to make concern the description of the entities of the system, as well as the relationships between them [8]. This decision making process can be supported by efficient optimization method proposed later in this paper.

## 3. Multiple UAVs System Architecture

**Figure 2:**
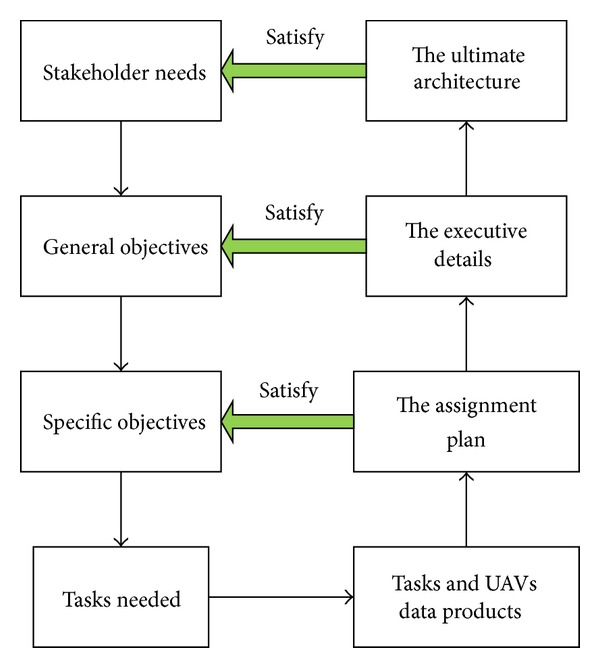
Architecture model for multi-UAVs system.

Based on the basic definition of system architecture, the description of multiple UAVs system architecture can be given. The process model for multiple UAVs system architecture is presented in Figure 2.

It can be extracted from the basic system architecture theory that the first step is identifying the stakeholder needs. The architecture of multiple UAVs system is designed to accomplish the value delivery loop based on the stakeholder needs, which is maximizing the mission performance given. Then, the second step is identifying how the multi-UAVs maximize the mission performance. This is the issue in the mission level. The embodiment of this is maximizing the probability of accomplishing all the tasks necessary effectively. In order to reach this goal, the suitable UAVs should be chosen firstly. This answers “what the system is.” Then, the UAVs and the tasks are modelled as an asymmetric bipartite graph, which represents the assignment relationship between UAVs and tasks. The selecting and assignment process is the embodiment of system architecting in the context of maximizing the mission performance.

**Figure 3:**
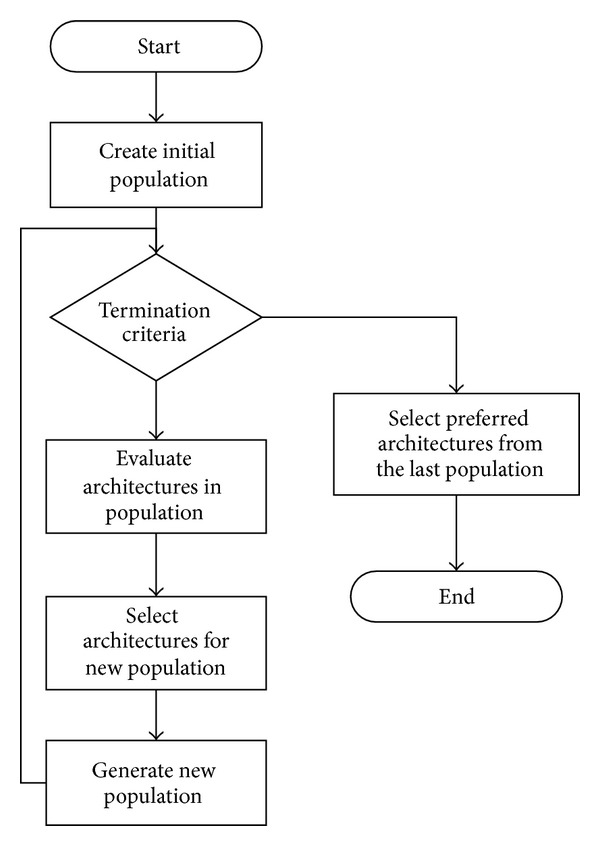
The general evolutionary process.

What is the architect trying to achieve? What makes an architecture “good?” To answer this question, a process is needed for selecting architectures that are not only capable of accomplishing the demand tasks but are also effective in terms of their overall value acquired. The quality of the proposed architecture is evaluated according to some criteria, which is the overall value acquired by the UAVs through conducting the tasks in this paper. It is inevitable for architect that the selecting and assigning should be done iteratively. The system architecting problem of multi-UAVs can be formulated as a constrained, combinatorial optimization problem. For example, these problems can be formulated as generalized assignment problems, quadratic assignment problems, or their expansions. As excellent population-based search algorithms, evolutionary algorithms (e.g., genetic algorithms, particle swarm optimization) can be chosen to solve these multi-UAVs system architecting problems. The general process of solving multi-UAVs system architecting problems employing evolutionary algorithms is presented in Figure 3. An optimization problem and an efficient genetic algorithm with a refined crossover operator (GA-RX) are proposed to accomplish the architecting process iteratively in the rest of this paper.

## 4. Problem Formulation

### 4.1. Notations

 
*M*: Number of available UAVs
 
*V* = {*U*_1_, *U*_2_,…, *U*_*M*_}: Set of all available UAVs
 
*N*: Number of tasks needed in mission
 
*T* = {*T*_1_, *T*_2_,…, *T*_*N*_}: Set of all tasks
 
*K*: Number of assets needed to be protected
 
*G*_*k*_: The set of tasks aimed for asset *k*, *k* = 1,2,…, *K*
 
*n*_*k*_: Number of tasks aimed for asset *k*, (i.e., |*G*_*k*_|), *k* = 1,2,…, *K*
 
*W* = [*W*_1_, *W*_2_,…, *W*_*K*_]: Value vector of assets, where *W*_*k*_ is the value of asset *k*
 
*P* = [*p*_*ij*_]_*N*×*M*_: The probability that UAV *j* accomplish task *i*
 
*π*_*i*_: The damage probability of the asset if the required tasks are not accomplished.

The decision variables will be denoted by *X* = [*x*_*ij*_]_*N*×*M*_;

(1) $$\begin{array}{c} x_{ij}=\{\begin{array}{cc} 1 & \text{if UAV}\;j\;\text{assigned to task}\;i\\ 0 & \text{otherwise.} \end{array} \end{array}$$

### 4.2. Objective Function and Constraints

*Objective Function*. The probability of that task *i* is accomplished is given as follows:

(2) $$\begin{array}{c} 1-\overset{M}{\underset{j=1}{\prod }}{\left(1-p_{ij}\right)}^{x_{ij}}. \end{array}$$

Therefore, the probability that asset *k* is protected successfully, that is, without being attacked by all targets, is given by

(3) $$\begin{array}{c} \underset{i\in G_{k}}{\prod }\left[1-\pi_{i}\overset{M}{\underset{j=1}{\prod }}{\left(1-p_{ij}\right)}^{x_{ij}}\right]. \end{array}$$

Hence, the objective function is given by

(4) $$\begin{array}{c} J=\overset{K}{\underset{k=1}{\sum }}W_{k}\underset{i\in G_{k}}{\prod }\left[1-\pi_{i}\overset{M}{\underset{j=1}{\prod }}{\left(1-p_{ij}\right)}^{x_{ij}}\right]. \end{array}$$

*Constraints*. The constraint in this problem is due to the fact that each UAV can be assigned to only one target because of the emergent property of the mission. The constraint is represented as follows:

(5) $$\begin{array}{c} \overset{N}{\underset{i=1}{\sum }}x_{ij}=1,\;j=1,2,\ldots ,M. \end{array}$$

### 4.3. Overall Problem Representation

The multiple UAVs system architecting problem can be stated as

(6) $$\begin{array}{c} \underset{\left\{x_{\mathrm{ij}}\in \left\{0,1\right\}\right\}}{\mathrm{Max}}J=\overset{K}{\underset{k=1}{\sum }}W_{k}\underset{i\in G_{k}}{\prod }\left[1-\pi_{i}\overset{M}{\underset{j=1}{\prod }}{\left(1-p_{ij}\right)}^{x_{ij}}\right] \end{array}$$

subject to ∑_*i*=1_^*N*^ *x*_*ij*_ = 1, *j* = 1,2,…, *M*.

## 5. Optimization Algorithm Design

### 5.1. General GA

Evolutionary algorithms (EAs) inspired by Darwinian principles of evolution are global stochastic search methods simulating the evolution process in nature. Among the EAs, GAs are most popular. GAs were introduced by Holland [9] and were applied to many practical problems by Goldberg [10]. GAs are of high availability, because the properties such as differentiability and continuity are not necessary. On the other hand, GAs are population-based optimization methods, which guarantee GAs effective approaches in searching for the global optimum. The general GA is shown in Pseudocode 1, where *P*(*t*) and *C*(*t*) are parents and offspring in generation *t*.

**Pseudocode 1:**
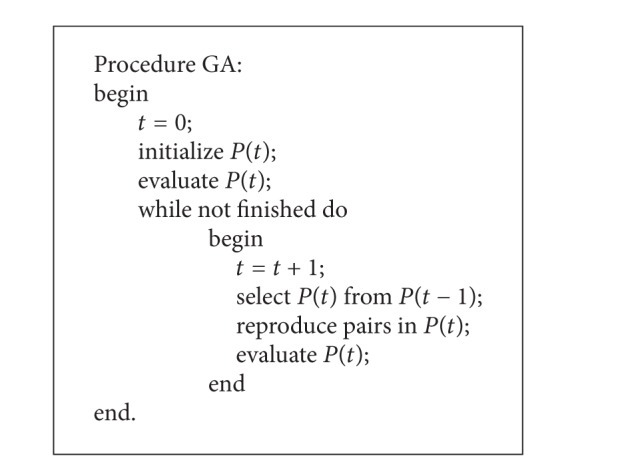
Pseudocode for GA.

### 5.2. Population Representation (Encoding) and Initialization

Genetic algorithms work with a number of potential solutions that are defined by a genotype representation according to the real solutions for certain problem. Encoding is the process of transformation from phenotype (in solution space) to genotype (in search space handled by GAs), decoding vice versa. The notion of real-valued genetic algorithms has been offered but is really a misnomer because it does not really represent the building block theory that was proposed by John Henry Holland in the 1970s. So we chose binary representation in this paper.

From the formulation given in problem formulation, we can extract the original formulation of the solution as follows:

(7) $$\begin{array}{c} \text{Solution}=\left(\begin{array}{ccc} x_{11} & \cdots & x_{1M}\\ \vdots & \ddots & \vdots \\ x_{N1} & \cdots & x_{NM} \end{array}\right),\\ x_{ij}=\{\begin{array}{cc} 1 & \text{if UAV}\;j\;\text{assigned to task}\;i\\ 0 & \text{otherwise.} \end{array} \end{array}$$

In any column (*x*_1*j*_,*x*_2*j*_,…,*x*_*Nj*_)^*T*^, the sole task assigned to the UAV *j* can be identified. The index of task assigned to UAV *j* is the *j*th bit of the interim integer string. Then, every bit of the interim integer string is transformed to its binary form. An example is given as follows.

Assume there are 5 UAVs and 3 tasks, and the original matrix *X* is

(8) $$\begin{array}{c} \left(\begin{array}{ccccc} 1 & 0 & 1 & 0 & 0\\ 0 & 1 & 0 & 0 & 1\\ 0 & 0 & 0 & 1 & 0 \end{array}\right). \end{array}$$

Its interim integer string is (1, 2, 1, 3, 2). Then the binary form which is called genotype is $\left(\begin{array}{ccccc} 01 & 10 & 01 & 11 & 10 \end{array}\right)$.

The merit of this representation is embodied as follows. (1) The constraint is eliminated, so it need not be handled additionally. (2) According to schema theory, binary representation is more beneficial to the growth of useful schemata, which in turn enhances convergence rates.

Having decided on the representation, the first step in the GA-RX is to create an initial population. We will adopt the method which is most usually achieved by generating the required number of individuals using a random number generator that uniformly distributes numbers in the desired range.

### 5.3. Genetic Operators Setup

In Pseudocode 1, it can be seen that the genetic operators included in GAs are Selection Operator, Crossover Operator, and Mutation Operator. In this paper, a refined crossover operator is proposed, while for the other genetic operators, the setup is as follows.

The desire for efficient selection methods is motivated by the need to maintain GAs overall time complexity. In this paper, the classical roulette wheel selection method based operator is selected because it provides zero bias and the spread is lower bounded.

In natural evolution, mutation is a random process where one allele of a gene is replaced by another to produce a new genetic structure. Binary mutation operator is selected for the reason that binary representation is selected.

A refined crossover operator will be introduced in the next section.

## 6. A Refined Crossover Operator

### 6.1. Origination

Crossover is the basic operator for producing new individuals in the GAs, and it is the most significant factor as for the effectiveness of certain GA. In traditional GAs, the crossover operator is executed between two individuals randomly extracted from *P*(*t*), who are selected from *P*(*t* − 1) using the selection operator (roulette wheel selection method based operator in this paper). More research is focused on the pattern of crossover, while little concerns the rules guiding the selecting of target individual to be operated.

Like its counterpart (marriage) in nature, in the process of crossover, an individual in the current *P*(*t*) always tends to be paired with another individual which is good enough. “Good enough” means the fitness of the partner selected is large enough. It would be best if its partner's fitness is the largest. This trend in the crossover can be easily comprehended using the mate selection of human.

### 6.2. Modeling and the Result Rule

Suppose that an individual in the current *P*(*t*) observes a sequence of *n* individuals whose fitness values are known. This individual who observes is the so-called administrator. The administrator has two choices for each randomly selected individual: accept or reject for crossover (like marriage). Once an individual is selected, the crossover will be executed between these two individuals, and the next individual as the individual that will be selected arrives randomly, the administrator can rank the applicant among all the individuals that arrived so far. However, please note that the administrator cannot select an individual that has not arrived yet even this individual has a high fitness. The question for the administrator individual is to discover the rule which can maximize the probability of selecting the best individual to execute crossover. This optimal policy problem has a strikingly elegant solution given by Vanderbei [11]. The optimal policy for the problem is a stopping rule. Under it, the administrator individual rejects the first *r* − 1 applicants (let applicant *M* be the best applicant among these *r* − 1 applicants) and then selects the first subsequent applicant that is better than applicant *M*. It can be shown that the optimal strategy lies in this class of strategies. For an arbitrary cutoff *r*, the probability that the best applicant is selected is

(9) $$\begin{array}{c} P\left(k\right)=\overset{N}{\underset{i=k+1}{\sum }}\frac{1}{N}\times \frac{k}{i-1}=\frac{k}{N}\overset{N}{\underset{i=k+1}{\sum }}\frac{1}{i-1}. \end{array}$$

Letting *n* tend to infinity, the limit of *r*/*n* is denoted by *x*, and using *t* for *i*/*n* and *dt* for 1/*n*, the sum can be approximated by the integral:

(10) $$\begin{array}{c} P\left(k\right)=x\overset{1}{\underset{x}{\int }}\frac{1}{t}dt=-x\ln x. \end{array}$$

Taking the differential coefficient of *P*(*x*) with respect to *x*, setting it to 0. By solving this equation, we can find that the optimal *x* is equal to 1/*e*. Thus, the optimal cutoff tends to *n*/*e*  (*e* ≈ 2.718) as *n* increases, and the best applicant is selected with probability 1/*e*  (37%).

### 6.3. Monte Carlo Simulation of the 1/*e*-Law

Monte Carlo experiment can be used to verify the effectiveness of the 1/*e*-law. The parameter settings of the Monte Carlo experiment are as follows.

1. The size of the applicant pool for selection, *n* is set to 20.
2. Computational times required are set to 10000.

**Figure 4:**
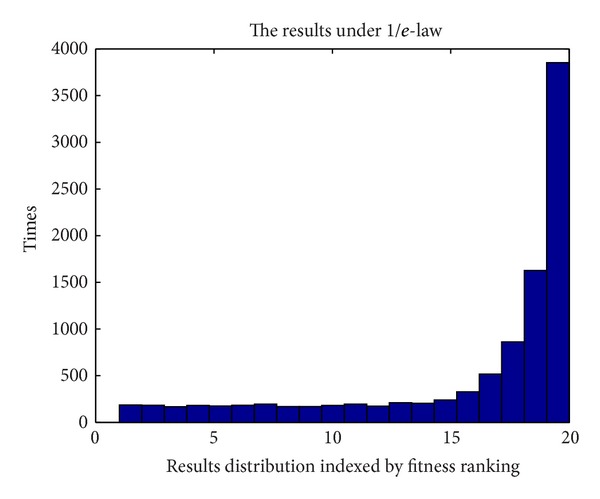
Simulation results.

The result is shown in Figure 4; the horizontal axis is the fitness ranking of individuals (as applicants in 1/*e*-law model) and the vertical axis is the statistical times (the total is 10000).

It can be seen that the best individual is selected with nearly 4000 times out of 10000 times, far more than other individuals. This shows the effectiveness of the 1/*e*-law in selection of best individual.

### 6.4. Refined Crossover Operator Based on 1/*e*-Law

Crossover operators used by the traditional genetic algorithm are common in selecting strategy. The parents are selected randomly, while in our novel algorithm an effective selecting strategy based on the 1/*e*-law is employed by the refined crossover operator. Firstly, the individuals produced by the selection process are ranked depending on their fitness values. Then, letting the first individual from the ranked population be the administrator, a sequence of up to *n* individuals is chosen randomly. The administrator rejects the first 37% individuals (let individual *X* be the best individual among these 37% individuals, whose fitness value is the largest in the first 37% individuals) and then selects the first subsequent individual that is better than individual *X*.

The two mating chromosomes, owned by the administrator individual and selected individual, are cut once at corresponding points and the sections after the cuts were exchanged. Here, a cross-site or crossover point is selected randomly along the length of the mated strings and bits next to the cross-sites are exchanged. A better solution can be obtained by combining good parents. A random recombination usually produce low quality solutions.

**Figure 5:**
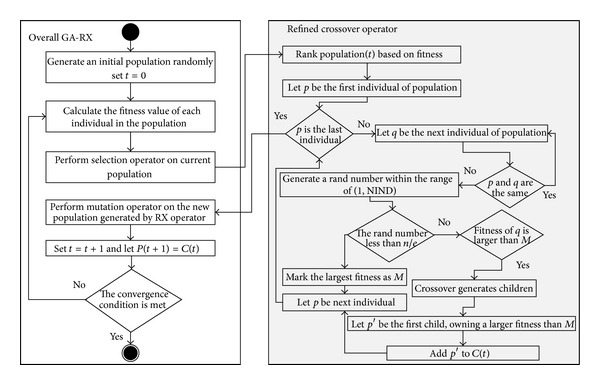
The overall process of GA-RX.

The pseudocode of RX operator is listed in Pseudocode 2, and the overall process of GA-RX is presented in Figure 5.

**Pseudocode 2:**
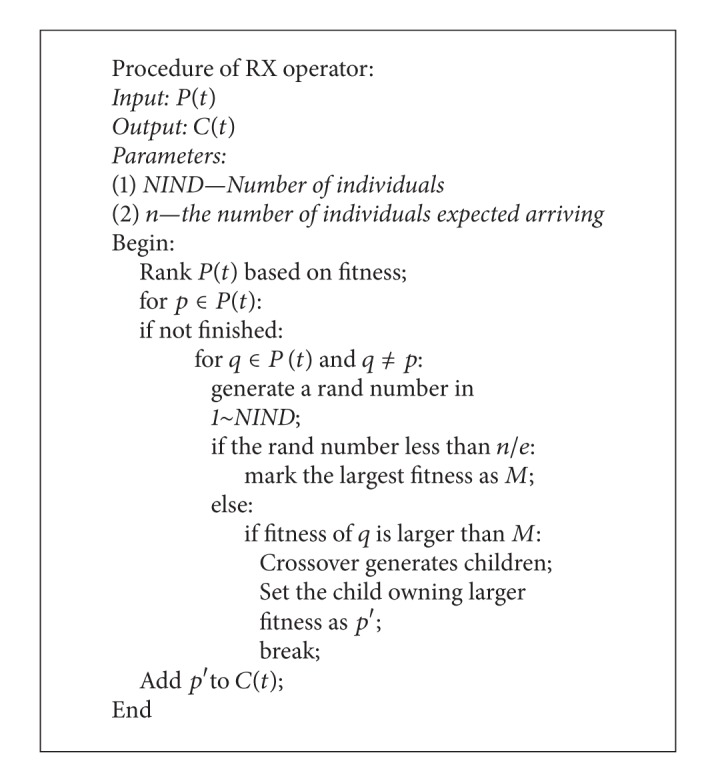
Pseudocode for RX Operator.

## 7. Simulations, Results, and Analysis

Two groups of simulations are executed, and two scenarios are considered.

### 7.1. Case Description

**Figure 6:**
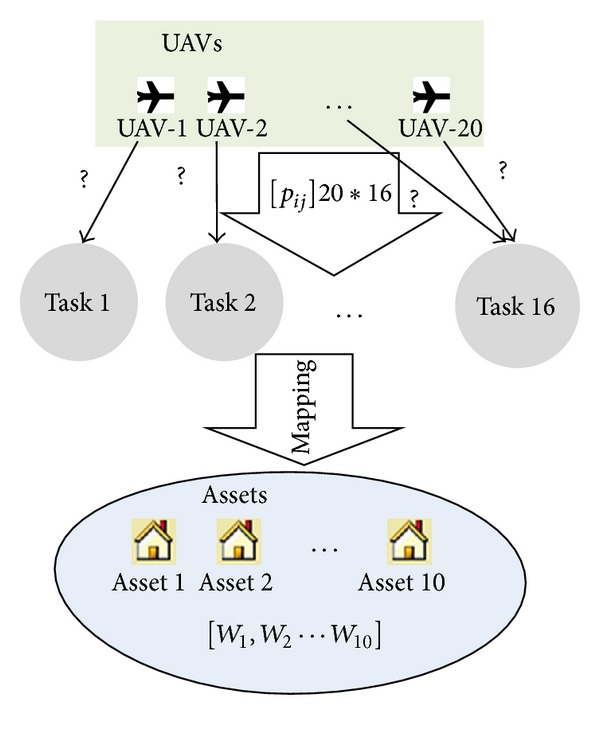
Scenario 1.

Two different scenarios are considered. The first scenario considers a simple situation which contains 20 UAVs, 16 tasks, and 10 assets (which are to be protected). The second scenario consists of data for 100 UAVs, 80 tasks, and 60 assets to be protected, which is considered as a complicated case for most of the state-of-the-art algorithms. For both scenarios, the following parameters are randomly generated, which are the value vector of assets (*W*), the probability matrix (*P* = [*p*_*ij*_]_*N*×*M*_), and the damage probability (*π*_*i*_). Both scenarios can be illustrated by Figure 6. For Scenario 1, the search space is 2^4×20^. A general evolutionary algorithm (e.g., GA, PSO, ACO, and DE) is sufficient to cope with such problems. However, for Scenario 2, the search space is significantly increased, that is, 2^7×100^. Intuitively, an advanced genetic operator is required such that the algorithm effectiveness and search efficiency can be improved when solving this system architecting problem.

The main reason for generating these data randomly is as follows: to the best of the authors' knowledge, there is no benchmark problem in the domain of system architecture optimization. Different studies employ different scenarios, objectives, and function formulations. In order to avoid bias on selected datasets and verify the effectiveness and efficiency of our proposed algorithm, that is, GA-RX, it is better to generate data randomly rather than choose a fixed scenario.

The performance of GA-RX is evaluated in two different ways. The first simulation is based on Scenario 1. For this case, a performance threshold is set, and the GA-RX is executed only for 50 generations (as previously mentioned, this is a simple example; 50 generations are sufficient for obtaining good results). This simulation is conducted using 100 groups of random data. An indicator, expressed as a percentage value, measures the number of times when the threshold performance is achieved over the total number of executions. Obviously, the larger the indicator is, the better the algorithm performance is. The second simulation is based on both scenarios. The GA-RX algorithm is compared with classical GA and three other state-of-the-art intelligent optimization algorithms, including CLPSO [12], DE [13], and ACO [14]. The relationship among these tasks is randomly identified in both scenarios. However, the employed data is identical for all simulations under the same scenario (which is to make a fair comparison). All simulations are performed 30 times. The performance of different algorithms is statistically compared using the *t*-test with a significance level of 95%.

### 7.2. Results

*Simulation 1*. For the 100 groups of data randomly generated, the times of the threshold performance met are 93, and the percentage is 93% (The threshold is 18).

*Simulation 2*. For simulation 2, the *t*-test results are in Table 1.

**Figure 7:**
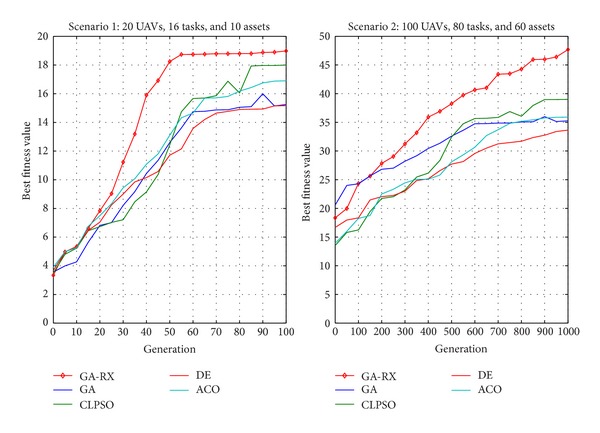
Typical results of simulation 2.

In the 30 times of each scenario, two typical simulation results are presented in Figure 7.

### 7.3. Analysis

Firstly, it can be observed that GA-RX implies a faster convergence rate than the other for algorithms in both scenarios. This is because the refined crossover operator derived from the 1/*e*-law can preserve the beneficial aspects of candidate solution while it does not destroy the diversity in each generation.

Secondly, Out of the five algorithms used for comparison, GA-RX is statistically better than all of the other algorithms. It can be observed that GA-RX can perform better than other algorithms in both scenarios. It results from the accumulation of refined crossover operator.

## 8. Conclusion

In this paper, the modeling and optimization of multi-UAVs system architecture alternatives have been studied. The multi-UAVs system architecture problem was presented as a constrained combinatorial optimization problem in Section 4. An enhanced genetic algorithm which employs a refined crossover operator (GA-RX) is proposed and applied to accomplish the architecting process of multiple UAVs system in Sections 5 and 6. The simulation results in Section 7 suggest that the GA-RX algorithm is available and effective in solving the multiple UAVs system architecture problem as it can preserve the beneficial aspects of candidate solution without degrading the diversity in each generation.

With respect to the future study, it is valuable to consider multiple objectives on the system architecture optimization [15]. Correspondingly, effective algorithms for solving these multi-objective optimization problems are expected to be designed [16, 17].

## Figures and Tables

**Table 1:** The *t*-test result of Simulation 2.

Scenario	Algorithm				
	GA	CLPSO	DE	ACO	GA-RX
	Mean/STD	Mean/STD	Mean/STD	Mean/STD	Mean/STD
Scenario 1	15.2/1.1	17.9/1.2	15.1/0.9	16.9/1.2	18.9/0.8
Scenario 2	35.2/8.7	39.0/5.1	33.6/4.7	35.9/6.8	47.7/3.5
